# Circulating tumour DNA in mCRPC: bridging tumour biology and real-time treatment monitoring

**DOI:** 10.3389/fmmed.2026.1852641

**Published:** 2026-07-31

**Authors:** Chimbabantu Kaoma, Mike Sathekge, Joseph Chalwe, Kgomotso Mokoala, Mankgopo Kgatle

**Affiliations:** 1 Department of Basic & Translational Research, Nuclear Medicine Research Infrastructure (NuMeRI), Pretoria, South Africa; 2 Department of Nuclear Medicine, University of Pretoria (UP) & Steve Biko Academic Hospital, Pretoria, South Africa; 3 Department of Medicine, University of Cape Town and Groote Schuur Hospital, Cape Town, South Africa

**Keywords:** androgen receptor pathway inhibitors, circulating tumor DNA, liquid biopsy, metastatic castration-resistant prostate cancer, PARP inhibitors, PSMA radioligand therapy, taxane chemotherapy

## Abstract

Metastatic castration-resistant prostate cancer (mCRPC) is associated with poor prognosis. Currently, treatment selection and response assessment in mCRPC still rely largely on prostate-specific antigen (PSA) and imaging tools, which may be insensitive and biologically noninformative. Liquid biopsy (LB), particularly circulating tumor DNA (ctDNA), offers a minimally invasive and reproducible approach for real-time assessment of tumor burden and tumor genomics. Across major treatment classes, ctDNA tumor fraction has shown consistent prognostic value, while selected genomic alterations may provide therapy-specific predictive information. Early on-treatment ctDNA kinetics are also emerging as dynamic markers of response, although prospective validation is required before routine clinical implementation. In this narrative review, we synthesize current evidence on ctDNA biology, detection methods, and its clinical utility across key mCRPC treatment classes, including androgen receptor pathway inhibitors (ARPIs), taxane chemotherapy, PARP inhibitors, and PSMA-targeted radioligand therapy (RLT). We critically examine ctDNA tumor fraction as a pan-therapy prognostic biomarker, genomic alterations as potential predictors of treatment sensitivity or resistance, early kinetic changes as surrogate markers of response, and the biological and technical limitations that currently restrict clinical adoption. Overall, the current evidence base for ctDNA in mCRPC remains predominantly prognostic rather than definitively predictive. Its integration into routine clinical practice will require prospective biomarker-driven trials, assay standardization, and a clear demonstration of clinical utility. At present, ctDNA is best positioned as a complementary tool for risk stratification and disease monitoring, with the potential to evolve into a clinically actionable biomarker for treatment decision-making.

## 1 Introduction

Metastatic castration-resistant prostate cancer (mCRPC) represents an advanced stage of prostate cancer (PCa) and remains associated with substantial morbidity and mortality despite significant therapeutic advances (Sung et al., 2021; Attard et al., 2016; Siegel et al., 2017). Over the past decade, multiple life-prolonging therapies, including androgen receptor pathway inhibitors (ARPIs), taxane-based chemotherapy, poly (ADP-ribose) polymerase inhibitors (PARPi), and prostate-specific membrane antigen (PSMA)-targeted radioligand therapy (RLT), have reshaped the treatment landscape (Hatano and Nonomura, 2023). However, optimal sequencing, patient selection, and early response assessment remain major clinical challenges, as robust, clinically validated biomarkers remain limited.

Current monitoring strategies rely primarily on prostate-specific antigen (PSA) kinetics, conventional imaging, and, more recently, PSMA PET/CT (Kinsella et al., 2018; Roessler et al., 2025; Hofman et al., 2020). While clinically useful, PSA lacks specificity for tumor biology, may not reflect clonal evolution, and can be discordant with radiographic progression (Mollica et al., 2020; Ignatiad et al., 2021). Although imaging is indispensable for disease detection and response assessment, it has inherent limitations. Conventional anatomical imaging depends primarily on size-based criteria, limiting its sensitivity for sub-centimeter metastatic disease (Hövels et al., 2008). PSMA PET has improved lesion detection and plays an important role in selecting patients for PSMA-targeted RLT; however, PSMA expression may be heterogeneous, and false-positive uptake can occur (Osman et al. 2021; Pontes et al., 2023; Grünig et al., 2021). Moreover, these modalities may not adequately capture early molecular alterations that underlie therapeutic resistance (Padhani et al., 2017). These limitations support the need for complementary biomarkers that provide real-time insight into tumor burden, molecular heterogeneity, and emerging resistance mechanisms.

Circulating tumor DNA (ctDNA), released into the bloodstream through tumor cell apoptosis, necrosis, and active secretion, offers a dynamic window into the evolving genomic landscape of mCRPC (Schwarzenbach et al., 2011; Otandault et al., 2020; Kustanovic et al., 2019). Advances in next-generation sequencing (NGS) and digital error-suppression techniques have enabled sensitive detection of tumor-derived alterations, including copy number changes, single-nucleotide variants, structural rearrangements, and tumor fraction metrics (Xu et al., 2017; Newman et al., 2016; Kinde et al., 2011). Unlike liquid biopsy (LB) assays primarily validated for early detection, pre-biopsy risk stratification, or repeat-biopsy decision-making (Matuszczak et al., 2021), ctDNA has particular relevance in advanced disease because it enables serial, non-invasive assessment of tumor genomics, clonal evolution, and treatment resistance.

In this review, we propose a structured framework in which ctDNA is evaluated across three complementary domains: quantitative tumor fraction as a marker of disease burden and biological aggressiveness; qualitative genomic alterations as potential predictors of treatment sensitivity or resistance; and longitudinal ctDNA kinetics as dynamic indicators of therapeutic response or futility. Distinguishing these roles is critical to avoid conflating prognostic associations with validated predictive utility (Kwan et al., 2022; Conteduca et al., 2017; Tolmeijer et al., 2023; Fonseca et al., 2024).

## 2 CTDNA biology and detection

ctDNA represents the tumor-derived fraction of total circulating cell-free DNA (cfDNA) present in plasma. In patients with metastatic cancer, cfDNA originates from both malignant and non-malignant cells; therefore, accurate interpretation requires an understanding of ctDNA biology, release mechanisms, and analytical constraints. In PCa, ctDNA should also be considered within the broader LB landscape, which includes circulating tumor cells (CTC), ctDNA, RNA, extracellular vesicles, exosomes, protein-based serum biomarkers, and genetic/epigenetic alterations in body fluids. This broader biological context is important because PCa is highly heterogeneous, and no single biomarker fully captures tumor burden, aggressiveness, spatial heterogeneity, or treatment resistance (Matuszczak et al., 2021).

### 2.1 Biological origin and release mechanisms

cfDNA was first documented in 1948 (Mandel, 1948). In healthy individuals, cfDNA is typically present at low concentrations and consists mainly of short fragments of approximately 40–200 bp, generated through chromatin cleavage during physiological apoptosis, predominantly from hematopoietic cells, including white blood cells and erythrocyte progenitors (Grabuschnig et al., 2020). In patients with cancer, a variable proportion of cfDNA is tumor-derived and is referred to as ctDNA. ctDNA enters the circulation through apoptosis, necrosis, mitotic catastrophe, therapy-induced cell death, and possibly active secretion *via* extracellular vesicles (Schwarzenbach et al., 2011; Kustanovic et al., 2019). The proportion of ctDNA within total cfDNA, known as the tumor fraction, is influenced by tumor burden, proliferative activity, genomic instability, treatment pressure, and metastatic site distribution, particularly visceral and bone involvement (Fonseca et al., 2024). Highly proliferative, poorly differentiated, genomically unstable, or treatment-resistant clones are more likely to shed detectable ctDNA than indolent or low-volume lesions. This explains why ctDNA is more frequently detectable in advanced mCRPC than in localized PCa, where tumor shedding may be low and intermittent. In earlier disease settings, where plasma ctDNA is often insufficient, non-ctDNA LB assays incorporating PCA3, TMPRSS2, HOXC6, DLX1, KLK3, and exosomal RNA markers may provide complementary diagnostic or risk-stratification information (Matuszczak et al., 2021).

ctDNA reflects a dynamic equilibrium between tumor cell turnover and systemic clearance mechanisms (Grabuschnig et al., 2020). Hepatic and renal elimination, nuclease-mediated degradation, and macrophage-driven clearance collectively account for its short half-life of approximately 1–2 h, enabling near real-time assessment of tumor dynamics. In advanced mCRPC, tumor fraction can range from less than 1% to more than 50%, reflecting differences in tumor burden and metastatic distribution (Fonseca et al., 2024; Conteduca et al., 2025; Kohli et al., 2020). Large-scale clinical analyses have further shown that ctDNA quantity varies significantly with patient age, sex, disease stage, and tumor type, influencing cfDNA yield and the likelihood of generating clinically useful reports (Huang et al., 2021).

**FIGURE 1:**
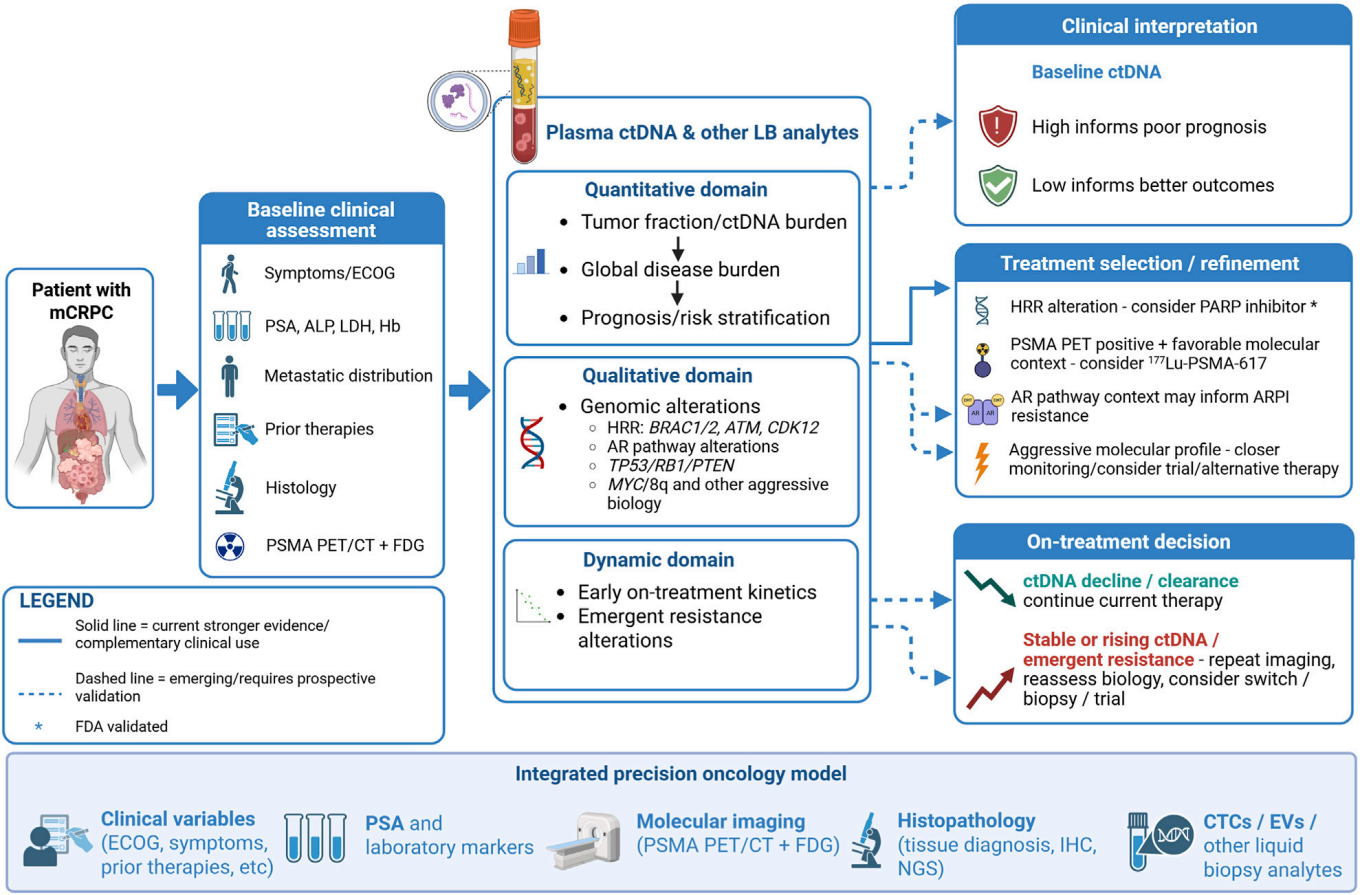
Clinical and translational roles of ctDNA in metastatic castration-resistant prostate cancer. ctDNA provides three complementary domains of information: quantitative tumor fraction for prognostic stratification, qualitative genomic alterations that may inform treatment sensitivity or resistance, and longitudinal kinetics for monitoring response and emerging resistance. These data should be integrated with clinical assessment, laboratory biomarkers, imaging, histopathology, and other liquid-biopsy analytes. Solid arrows represent established or better-supported applications, whereas dashed arrows indicate emerging applications requiring prospective validation. The asterisk denotes a regulatory-validated application. Created with BioRender.com.

In mCRPC, ctDNA analysis provides a non-invasive approach for genomic profiling, assessment of tumor burden, longitudinal monitoring of treatment response, detection of emerging resistance mechanisms, and prognostic stratification. High tumor fraction has consistently been associated with adverse clinical outcomes across treatment modalities, suggesting that ctDNA burden primarily reflects global disease biology rather than therapy-specific sensitivity or resistance. The principal clinical and translational applications of ctDNA in mCRPC are summarized in Figure 1.

### 2.2 Genomic composition

ctDNA encompasses diverse classes of genomic alterations relevant to PCa biology, including copy number changes such as *AR* amplification, *AR* mutation, single-nucleotide variants, and small insertions or deletions in genes like *TP53* and *BRCA2*, structural rearrangements and gene fusions, as well as genome-wide measures of aneuploidy and genomic instability (Tan et al., 2022; Tukachinsky et al., 2021). These alterations are biologically meaningful because they reflect key hallmarks of advanced PCa, including persistent AR pathway dependence, defective DNA repair, lineage plasticity, and loss of tumor suppressors. However, broader LB literature emphasizes that clinically useful biomarkers in PCa are not limited to DNA sequence alterations but can also be assessed across multiple molecular dimensions, including, CTC, transcriptional dysregulation, protein processing, epigenetic modifications, and extracellular vesicle biology (Matuszczak et al., 2021; Antonarakis et al., 2016).

Because ctDNA represents a composite signal derived from multiple metastatic sites, it offers a broader view of spatial heterogeneity than a single-site tissue biopsy. This is particularly relevant in mCRPC, where resistant subclones may differ between metastases. Nevertheless, ctDNA analysis cannot resolve intratumoural architecture, histological transformation, tumor microenvironmental context, or site-specific biology. Therefore, ctDNA is best interpreted as a systemic genomic signal rather than a replacement for tissue histology, imaging, or clinical assessment.

### 2.3 Clonal evolution under therapeutic pressure

mCRPC is characterized by ongoing clonal evolution driven by AR signaling, DNA damage repair defects, and lineage plasticity (Kohli et al., 2020; Imamura et al., 2023; Beltran et al., 2016). Serial ctDNA sampling enables longitudinal assessment of subclonal dynamics and resistance-associated alterations, including AR amplification, ligand-binding domain (LBD) mutations, *BRCA2* reversion alterations, *PTEN* loss, *TP53* disruption, *RB1* loss, and PI3K–AKT pathway aberrations (Kohli et al., 2020; Tripathi et al., 2023). This capacity to monitor tumor evolution over time distinguishes ctDNA from static archival tissue biopsies and supports its potential to detect molecular progression before radiographic change.

Despite castration, most mCRPC tumors remain dependent on AR signaling. Under selective pressure from androgen deprivation therapy (ADT) and AR pathway inhibitors, gain-of-function *AR* mutations accumulate and sustain receptor activity despite low androgen levels. Many of these alterations occur within the LBD and broaden ligand specificity, permitting activation by adrenal steroids, progesterone, glucocorticoids, or antiandrogens. Representative mutations include T878A, H875Y, L702H, and F877L, which contribute to persistent AR signaling and therapeutic resistance (Shi et al., 2002; Quistini et al., 2025; Karantanos et al., 2013).

Certain gain-of-function mutations directly mediate resistance to contemporary AR signaling inhibitors. F877L can convert enzalutamide and apalutamide from antagonists to agonists, whereas L702H enables AR activation by glucocorticoids administered during systemic therapy, illustrating therapy-driven selection of resistant subclones (Quistini et al., 2025; Doamekpor et al., 2023; Gao, 2023).

In contrast, loss-of-function AR alterations are uncommon and appear to contribute less to resistance than lineage plasticity and epigenetic reprogramming. Reduced AR signaling is more frequently associated with neuroendocrine differentiation and AR-indifferent states, allowing tumors to evade AR-directed therapies through alternative oncogenic programs (Imamura et al., 2023; Shiota et al., 2022; Storck et al., 2022).

From a cancer biology perspective, AR alterations exemplify Darwinian evolution under therapeutic selection. Sequential exposure to ADT and AR-targeted agents promotes the emergence of heterogeneous subclones harbouring both AR-dependent and AR-independent resistance mechanisms, contributing to intrapatient heterogeneity, metastatic progression, and treatment failure (Gao, 2023; Brooke and Bevan, 2009).

Longitudinal ctDNA studies demonstrate that AR pathway inhibition induces greater subclonal selection and evolutionary restructuring than taxane chemotherapy, with resistant clones frequently enriched for PI3K–AKT pathway alterations. Moreover, matched tissue and liquid biopsy analyses show that the genomic complexity of metastatic prostate cancer increases with treatment exposure, emphasizing that therapy itself is a major driver of tumor evolution (Zhao et al., 2025).

From a companion diagnostics perspective, detection of AR alterations in ctDNA offers a noninvasive approach for identifying resistance mechanisms, monitoring clonal evolution, and predicting response to AR-targeted therapies. Molecular assays capable of detecting clinically relevant AR variants may therefore facilitate treatment stratification and support precision medicine approaches in mCRPC (Tripathi et al., 2023; Martignano et al., 2016; Bernard-Tessier et al., 2023).

Overall, gain-of-function AR mutations maintain oncogenic AR signaling and drive resistance to hormonal therapies, whereas diminished AR dependence is more commonly associated with lineage plasticity and neuroendocrine differentiation. Integration of these evolutionary changes through ctDNA analysis provides important biological insights and supports the development of companion diagnostic strategies for precision treatment in mCRPC (Imamura et al., 2023; Gao, 2023; Storck et al., 2022).

### 2.4 Analytical platforms and detection sensitivity

Advances in NGS have enabled increased ctDNA detection through approaches such as targeted hybrid-capture panels, amplicon-based deep sequencing, digital droplet (dd) PCR for hotspot mutations, and low-pass whole-genome sequencing for tumor fraction estimation (Tébar-Martínez et al., 2023; Postel et al., 2018; Husain et al., 2022; Chen and Zhao, 2019). Incorporating molecular barcoding and error-suppression strategies can achieve variant-allele frequency (VAF) detection thresholds below 0.1% under optimized conditions. Sensitivity, however, remains dependent on the quantity of input DNA, sequencing depth, tumor fraction, and the robustness of bioinformatic pipelines.

Analytical sensitivity must be interpreted in the context of biological sensitivity. A technically sensitive assay may still fail to detect tumor-derived alterations when the tumor fraction is extremely low, when the disease is confined to sites with limited shedding, or when the relevant resistant clone is below the assay’s limit of detection. A non-detectable result, therefore, does not exclude tumor presence, clinically significant disease, or emerging resistance.

### 2.5 Biological and technical confounders

A major limitation in ctDNA interpretation is clonal hematopoiesis of indeterminate potential (CHIP), in which age-related somatic mutations in hematopoietic stem cells release mutated DNA into the plasma. Mutations in genes such as *TP53, ASXL1, DNMT3A*, and *TET2* may therefore originate from non-malignant clones rather than tumor tissue, making matched white blood cell sequencing essential to reduce false-positive attribution (Mayrhofer et al., 2018). Additional challenges include pre-analytical variability related to tube type, processing time, and storage conditions, as well as a lack of assay harmonization across platforms and the absence of universally validated tumor fraction thresholds for clinical decision-making.

Translational implementation is further constrained by population and clinical-context variability. Many LB assays have been validated in selected cohorts, and their performance across diverse ethnic groups, disease states, and treatment settings remains incompletely defined. As a result, cut-off values may not be directly transferable between populations or clinical scenarios. Although LB biomarkers hold substantial clinical promise, broad implementation will require rigorous validation, assay standardization, and context-specific interpretation.

### 2.6 Conceptual framework for clinical application

**FIGURE 2:**
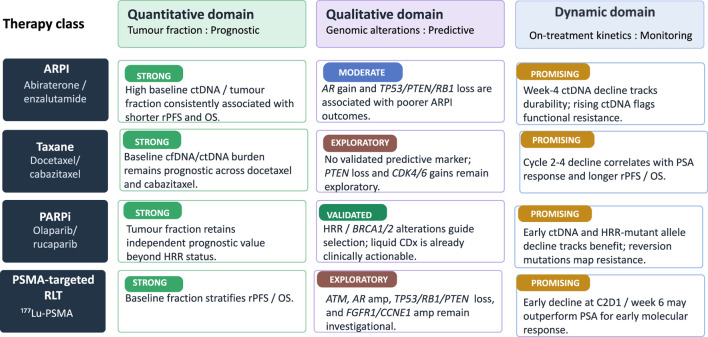
Conceptual framework for clinical application of ctDNA in metastatic castration-resistant prostate cancer. This figure illustrates the three complementary domains of ctDNA utility: (i) quantitative tumor fraction, serving as a prognostic marker of global disease burden and biological aggressiveness; (ii) qualitative genomic alterations, which may predict sensitivity or resistance to specific therapies such as PARP inhibitors or androgen receptor pathway inhibitors; and (iii) dynamic on-treatment kinetics, reflecting early molecular response or resistance during therapy. The framework summarizes the relative strength of current evidence supporting each domain across major treatment classes, including AR pathway inhibitors (ARPIs), taxane chemotherapy, PARP inhibitors, and PSMA-targeted radioligand therapy (RLT). Together, these domains highlight ctDNA’s potential as a multidimensional biomarker system for prognostication, therapy selection, and real-time monitoring in mCRPC. Created with BioRender.com.

In mCRPC, ctDNA should be defined by validated analytical performance, reproducible detection at clinically relevant sensitivity and specificity, and a clear link between the ctDNA finding and a treatment-relevant biological or genomic feature (Mateo et al., 2018; U.S. Food and Drug Administration, 2024). Based on current evidence, ctDNA biology in mCRPC can be organised into three clinically relevant domains (Figure 2): a quantitative domain, in which tumour fraction reflects global disease burden and prognosis; a qualitative domain, in which genomic alterations may inform sensitivity or resistance to therapies, including homologous recombination repair (HRR) defects relevant to PARP inhibitor use and AR pathway alterations associated with resistance to androgen receptor pathway inhibitors; and a dynamic domain, in which early on-treatment ctDNA kinetics may indicate response or futility (Mateo et al., 2018). Distinguishing these domains is essential to avoid conflating prognostic associations with validated predictive utility; therefore, ctDNA should guide treatment selection, escalation, de-escalation, or switching only when supported by prospective clinical validation with prespecified thresholds and analyses, and preferably by adequately powered interventional studies showing improvement in clinically meaningful outcomes beyond standard clinical, biochemical, imaging-based, and disease-context assessment (U.S. Food and Drug Administration, 2024).

## 3 CtDNA tumour fraction as a pan-therapy prognostic biomarker

Current evidence supports baseline ctDNA tumour fraction in mCRPC primarily as a prognostic, rather than predictive, biomarker: it identifies patients at higher risk of adverse outcomes largely irrespective of the treatment received, whereas a predictive biomarker should demonstrate differential benefit or harm from a specific therapy (Pabani and Gyawali, 2026; Ou et al., 2021). Higher ctDNA levels appear to capture quantitative disease burden and aggressive tumour biology and have been associated with poorer outcomes across therapeutic contexts, supporting their use for risk stratification rather than stand-alone treatment selection (Thapa et al., 2026). Accordingly, ctDNA tumour fraction should not be interpreted as evidence that one therapy is preferable to another unless the biomarker is shown to modify treatment effect in appropriately designed studies, preferably through prespecified subgroup or interaction analyses in adequately powered randomized trials (Ou et al., 2021; Duffy, 2022). This distinction is clinically important because conflating prognosis with prediction may justify premature escalation, switching, or withholding of therapy without evidence that acting on the ctDNA result improves outcomes (Pabani and Gyawali, 2026).

### 3.1 Androgen receptor pathway inhibitors

In ARPIs-treated cohorts, ctDNA positivity and increasing tumor fraction have been consistently associated with significantly shorter overall survival (OS) and radiographic progression free survival (rPFS), even after adjustment for clinical variables such as PSA, performance status, and metastatic distribution (Table 1). Landmark trials have shown that ctDNA-positive patients receiving potent AR-targeted therapies such as abiraterone or enzalutamide experience substantially worse outcomes, with an overall median survival reduced from 47.4 to 27.3 months and median rPFS from 33.0 to 17.8 months, independent of other risk factors (Knutson et al., 2024). In this study, each 10% increase in ctDNA aneuploidy fraction was associated with a proportional increase in the hazard of progression and death, underscoring a proportional relationship between ctDNA and adverse outcomes. These findings have been reinforced by other studies, such as TALAPRO-2, in which a high baseline ctDNA burden predicted poor outcomes across treatment arms, and early reductions in ctDNA fraction correlated with improved rPFS (Conteduca et al., 2017; Tolmeijer et al., 2023; Azad et al., 2024). Importantly, ctDNA dynamics also provide a more sensitive measure of resistance than PSA alone, with a persistent or rising ctDNA burden during ARPI therapy strongly predicting functional resistance (Tolmeijer et al., 2023).

**TABLE 1:** Key ctDNA-focused studies in ARPi-treated mCRPC.

Study name	Year	Study design	Sample size	Treatment context	Biomarker	Key findings
Conteduca et al. (2017)	2017	Correlative biomarker analysis with a validation cohort	171 patients +94 patients for validation	Abiraterone/Enzalutamide	*AR* copy number gain	AR amplification is associated with poor outcomes on ARPI therapy
Tolmeijer et al. (2020)	2020	Systematic review and meta-analysis	>1000 patients	ARPI and taxane therapy	*AR* copy number gain	*AR* gain predicts shorter PFS and OS on ARPI therapy
Tolmeijer et al. (2023)	2023	Prospective biomarker study	81 patients	First-line ARPI	ctDNA fraction kinetics	Early decline predicts improved survival and treatment response
Alliance A031201 (Knutson et al., 2024)	2024	Phase III randomized trial	1,311 patients	Enzalutamide ± Abiraterone	ctDNA positivity	ctDNA presence associated with inferior survival outcomes
TALAPRO-2 (Azad et al., 2024)	2024	Phase III randomized controlled trial	805 (all commers) + 230 (HRR-deficient)	ARPI + PARP inhibitor	Baseline ctDNA fraction	High baseline ctDNA fraction associated with worse clinical outcomes

Beyond tumor fraction, specific genomic alterations provide additional prognostic and potential predictive information. Gains in *AR* and *MYC/MYCN*, together with loss of *TP53, PTEN*, and *RB1,* identify molecular subsets with inferior outcomes (Quistini et al., 2025; Kostos et al., 2023; Tolmeijer et al., 2020; Visakorpi et al., 1995). This highlights the prognostic utility of ctDNA in ARPI therapy.

### 3.2 Taxane chemotherapy

In the taxane setting, ctDNA tumor fraction functions primarily as a prognostic and monitoring biomarker rather than as a validated predictive marker for treatment selection (Table 2). Large phase III trials such as FIRSTANA and PROSELICA demonstrated that higher baseline cfDNA and failure to reduce cfDNA at cycles 2 and 4 were independently associated with shorter rPFS and OS in men receiving docetaxel or cabazitaxel (Mehra et al., 2018). Cohort-level and pooled analyses further confirmed that elevated baseline ctDNA levels predict inferior survival outcomes across taxane-treated populations (Fonseca et al., 2024; Brighi et al., 2025). While exploratory studies suggest that alterations such as *PTEN* loss or *CDK4/6* amplification may be associated with poorer outcomes under chemotherapy (Fonseca et al., 2024; TOREN and ZOUBEIDI, 2014; Jamaspishvili et al., 2018; Kamisawa et al., 2022) Currently, no dominant molecular determinant of taxane resistance has been validated for clinical decision-making within this class. These findings underscore the broader principle that tumor fraction captures global disease biology rather than drug-specific sensitivity or resistance.

**TABLE 2:** ctDNA biomarkers in taxane-treated mCRPC.

Study name	Year	Study type	Sample size	Treatment	ctDNA metric	Key findings
Mehra et al. (2018)	2018	Biomarker analysis of Phase III trials (FIRSTANA, PROSELICA)	571 patients (2502 samples)	Docetaxel/Cabazitaxel	Baseline cfDNA concentration and early kinetics (3–9 weeks)	Baseline cfDNA is prognostic; early cfDNA decline is associated with PSA response and longer rPFS/OS
Liu et al. (2021)	2021	Systematic review and meta-analysis	23 studies	Mixed systemic therapy, including taxane-based cohorts	cfDNA concentration	High cfDNA concentration is associated with poorer PFS, with stronger associations reported in mCRPC
Ruiz-Vico et al. (2025)	2025	Biomarker substudy of Phase III RCT	157 patients	Docetaxel ± Enzalutamide	ctDNA burden and kinetics	High baseline ctDNA and persistence or rise of ctDNA after one cycle is associated with shorter PFS
Liu (2025)	2025	Meta-analysis	13 studies (1,946 patients)	Mixed therapies, including taxane-based cohorts	ctDNA fraction	High baseline ctDNA fraction predicts shorter PFS and OS

### 3.3 Poly (ADP-ribose) polymerase inhibitors

In the PARPi setting, HRR alterations, such as *BRCA1/2* mutations, are predictive of therapeutic sensitivity, with systematic reviews and meta-analyses confirming maximal benefit in BRCA-mutated mCRPC compared to those with *ATM* or other HRR defects (Table 3) (Alameddine et al., 2023; Wu et al., 2021). In the PROfound trial, ctDNA testing showed substantial concordance with tissue sequencing for *BRCA1*, *BRCA2*, and *ATM* alterations, supporting ctDNA as a complementary non-invasive approach for identifying patients for PARP inhibitor therapy, particularly when tissue is insufficient. These findings contributed to the regulatory approval of olaparib and to the FDA approval of FoundationOne CDx and FoundationOne Liquid CDx as companion diagnostics (Chi et al., 2023; Woodhouse et al., 2020). Beyond predictive utility, baseline ctDNA burden appears to retain prognostic value in this setting, with higher ctDNA fraction/positivity associated with inferior PFS and OS, underscoring that quantitative tumor load and qualitative genomic vulnerability represent biologically distinct domains (Kopytov et al., 2025; Chanhih et al., 2025). Exploratory studies such as TOPARP-A have shown that early reductions in ctDNA fraction and HRR-mutant allele frequency strongly predict longer rPFS and OS, whereas persistence or rise in ctDNA burden identifies non-responders and maps resistance mechanisms such as *BRCA* or *PALB2* reversion mutations (Mateo et al., 2015; Goodall et al., 2017). These findings highlight ctDNA as an important biomarker in PARPi therapy, capable of guiding patient selection, prognostication, monitoring treatment response, and detecting resistance. Therefore, it may complement conventional markers such as PSA or imaging (Kostos et al., 2023; Kopytov et al., 2025; Chanhih et al., 2025; Loehr et al., 2023; Yuan et al., 2022).

**TABLE 3:** Key ctDNA studies in PARPi-treated mCRPC.

Study name	Year	Study design	Sample size	Treatment	ctDNA biomarker	Key findings
TOPARP-A (Mateo et al., 2015)	2015	Phase II clinical trial	49 patients	Olaparib	HRR mutations detected in ctDNA	Showed improved responses in DDR/HRR defects (*BRCA2* and *ATM* aberrant tumours)
TOPARP-A (Goodall et al., 2017)	2017	Phase II clinical trial (Prospective biomarker analysis: serial cfDNA sequencing)	46/49 patients had serial cfDNA	Olaparib	Serial cfDNA sequencing and HRR mutations	≥50% fall in cfDNA at 4–8 weeks associated with improved rPFS/OS; detected *BRCA2*/*PALB2* reversion mutations as resistance mechanisms
PROfound (Woodhouse et al., 2020)	2020	Phase III randomised trial	387 patients (Cohort A 245; Cohort B 142)	Olaparib vs. ARPI	HRR mutations	Showed improved rPFS in HRR. Led to FDA approval of FoundationOne® Liquid CDx for olaparib selection
TRITON-2 (Abid et al., 2023)	2023	Phase II clinical trial	277 patients	Rucaparib	BRCA1/2 alterations	Clinical benefit of rucaparib in mCRPC BRCA mutation: BRCA reversion mutations showed a significant association with resistance to rucaparib

### 3.4 PSMA-targeted radioligand therapy

PSMA-targeted RLT with ^177^Lu-PSMA-617 has emerged as an effective treatment for selected patients with mCRPC. Unlike ARPIs or taxane chemotherapy, ^177^Lu-PSMA-617 delivers β-particle radiation directly to PSMA-expressing tumor cells, inducing DNA damage resulting in cell apoptosis (Hennrich and Eder, 2022; Zhao et al., 2023). PSMA-targeted PET serves as a companion diagnostic tool for PSMA-targeted RLT by confirming sufficient whole-body PSMA expression, assessing disease distribution, and identifying PSMA-negative lesions that may not respond to treatment. This may occasionally be used with FDG PET, which informs on the presence of metabolically active, aggressive, or dedifferentiated lesions that may show low or absent PSMA expression. The presence of FDG-positive/PSMA-negative discordant disease is clinically significant because such lesions are less likely to respond to PSMA-directed therapy and may indicate poor prognosis. PSMA and FDG PET are therefore essential for selecting patients suitable for ^177^Lu-PSMA-617 therapy (Sartor et al., 2021; Hofman et al., 2018). In contrast, ctDNA assays provide complementary molecular information, including tumor fraction, DNA repair defects, AR pathway alterations, and emerging resistance clones. While ctDNA can support prognostication, treatment sequencing, and monitoring of clonal evolution, it cannot replace PSMA PET because it does not localize disease or confirm lesion-level PSMA expression. Thus, PSMA PET and ctDNA should be viewed as complementary tools: PSMA PET determines eligibility for PSMA-directed therapy, whereas ctDNA refines molecular risk assessment and treatment monitoring.

#### 3.4.1 Quantitative domain: tumor fraction as a prognostic biomarker

Consistent with observations across ARPI, taxane, and PARP inhibitor cohorts, baseline ctDNA tumor fraction demonstrates a reproducible, therapy-agnostic prognostic effect in patients undergoing ^177^Lu-PSMA-617 therapy. In the PSMAfore trial, an elevated baseline ctDNA fraction (>0.5%) was independently associated with inferior rPFS, irrespective of treatment assignment, reinforcing tumor fraction as a global marker of adverse disease biology rather than a predictor of RLT-specific sensitivity (De Bono et al., 2025). Similarly, exploratory analyses from TheraP showed that patients with very low baseline ctDNA fraction (<2%) experienced higher PSA response rates and prolonged survival following ^177^Lu-PSMA-617 (Kwan et al., 2025). These findings align with broader mCRPC literature, indicating that tumor fraction integrates metastatic burden, proliferative rate, and clonal diversity. From a biological perspective, since RLT induces tumor cell death through β-particle-mediated DNA damage, overall tumor burden, clonal heterogeneity, and intrinsic DNA repair capacity may critically influence therapeutic efficacy. High ctDNA tumor fraction likely reflects extensive metastatic volume and evolutionary fitness, factors that may dilute effective absorbed radiation dose per lesion and increase the probability of resistant subclonal populations. Importantly, these associations appear prognostic rather than definitively predictive as elevated tumor fraction identifies patients with biologically aggressive disease across treatment modalities, including in RLT.

#### 3.4.2 Dynamic domain: early ctDNA kinetics during RLT

Given the short half-life of ctDNA (about 1–2 h), early on-treatment changes offer a biologically plausible indicator of treatment effect in radiation-based therapies. In PSMAfore, ctDNA fraction measured at cycle 2 days 1 (C2D1) and early reductions in tumor fraction correlated more strongly with rPFS and OS than baseline levels and were largely independent of PSA kinetics (De Bono et al., 2025). In TheraP, early declines in ctDNA were similarly associated with favorable outcomes in patients treated with ^177^Lu-PSMA-617 (Kwan et al., 2025). These findings suggest that ctDNA kinetics may reflect early molecular response to radiation-induced DNA damage before anatomical changes become radiographically apparent. However, clinically actionable thresholds for defining meaningful decline have not been standardized, and prospective validation in biomarker-driven RLT trials is required before routine clinical implementation. At present, ctDNA kinetics should be considered investigational but highly promising as an early monitoring tool.

#### 3.4.3 Qualitative domain: genomic alterations and response heterogeneity

Beyond quantitative burden, ctDNA profiling enables interrogation of genomic features that may influence RLT response. Unlike PARP inhibitor therapy, where HRR defects have validated predictive utility, genomic correlates in RLT cohorts remain largely investigational.

##### 3.4.3.1 DNA damage repair pathways and radiosensitivity

Because β-particle radiation induces DNA damage, intrinsic DNA repair competency is biologically relevant. In TheraP, *ATM* alterations were associated with greater relative benefit from ^177^Lu-PSMA-617 compared with cabazitaxel, suggesting a potential interaction between impaired DNA damage repair and radiation-induced cytotoxicity (Kwan et al., 2025). *ATM* loss disrupts DNA repair signaling and may enhance radiosensitivity, providing a mechanistic rationale for potential predictive enrichment. While compelling, these observations remain exploratory and require prospective validation to establish whether DNA repair alterations can guide RLT selection.

##### 3.4.3.2 AR pathway aberrations

AR signaling modulates PSMA expression at the transcriptional level (Vaz et al., 2020). AR pathway activation has been associated with reduced PSMA expression in preclinical models, whereas AR blockade can upregulate PSMA. In clinical RLT cohorts, AR amplification detected *via* ctDNA has been associated with inferior outcomes following ^177^Lu-PSMA-617 (Vanwelkenhuyzen et al., 2023; De Giorgi et al., 2021). However, these findings likely reflect a combination of aggressive tumor biology, lineage plasticity, and potential heterogeneity in PSMA target expression rather than a validated mechanism of resistance. Although AR signaling regulates PSMA expression, the association between specific AR mutations and PSMA expression remains investigational; therefore, ctDNA-defined AR alterations should be interpreted alongside, rather than in place of, PSMA PET imaging for assessing target availability before RLT.

##### 3.4.3.3 Tumor suppressor gene alterations and aggressive variant biology

Alterations in *TP53*, *RB1*, and *PTEN* genes are frequently associated with aggressive-variant prostate cancer (AVPC) and lineage plasticity. These have been shown to be enriched in poor responders to RLT in exploratory analyses (Crumbaker et al., 2023; Beltran et al., 2019). These alterations may mark tumors with high proliferative capacity, genomic instability, and phenotypic heterogeneity, factors that could reduce the likelihood of durable radiation response. Again, these associations are best interpreted as prognostic signals reflecting aggressive biology rather than validated predictive biomarkers of RLT resistance.

##### 3.4.3.4 Oncogene amplifications

Copy number gains such as *MYC*/8q have been associated with inferior outcomes in PSMAfore, particularly when co-occurring with high tumor fraction (De Bono et al., 2025). Additional exploratory series have identified amplifications in genes, including *FGFR1* and *CCNE1,* among non-responders (Sartor et al., 2023). These alterations implicate proliferative and cell-cycle regulatory pathways as candidate contributors to treatment failure, although prospective confirmation is required.

##### 3.4.3.5 Acquired resistance and phenotypic plasticity

Longitudinal ctDNA analyses from TheraP and LuPIN have not identified a single recurrent genomic alteration consistently accounting for RLT progression, suggesting that resistance is multifactorial (Kwan et al., 2025; Crumbaker et al., 2023). Beyond mutation-based mechanisms, resistance may involve epigenetic reprogramming, PSMA downregulation, or lineage plasticity, including neuroendocrine transformation, processes that may not be fully captured by targeted sequencing alone (Lyma et al., 2025). Emerging methylation-based ctDNA assays and fragmentomic approaches have demonstrated the capacity to detect neuroendocrine differentiation and other phenotypic shifts in mCRPC (Franceschini et al., 2024; Berchuck et al., 2022). These platforms may complement mutation-based assays by capturing transcriptional reprogramming and epigenetic adaptation under radiation pressure. However, their role in guiding RLT remains investigational.

Collectively, the current evidence supports a multidimensional, hierarchically weighted role for ctDNA in the context of ^177^Lu-PSMA-617 therapy (Table 4). Within this framework, quantitative tumor fraction emerges as the most consistently validated domain, providing reproducible prognostic stratification across RLT cohorts. In contrast, early on-treatment kinetic changes represent a promising dynamic indicator of molecular response, reflecting the biological impact of radiation-induced DNA damage before radiographic progression becomes apparent. Selected genomic alterations, particularly those involving DNA damage repair pathways and key tumor suppressor genes, may further contribute to interpatient response heterogeneity; however, these associations remain exploratory and lack prospective validation as predictive biomarkers. Importantly, most genomic correlates identified in RLT studies should be interpreted as investigational rather than treatment-defining. Future precision oncology paradigms are likely to integrate ctDNA-derived measures of tumor burden and genomic vulnerability with functional PSMA PET imaging parameters, enabling composite models that incorporate molecular load, target expression, and DNA repair competency to refine patient selection and risk stratification. Until such biomarker-driven strategies are validated in prospective, stratified clinical trials, ctDNA in the RLT setting should be regarded primarily as a robust prognostic tool with emerging monitoring capabilities, rather than a definitive predictive biomarker for therapeutic decision-making.

**TABLE 4:** Emerging ctDNA biomarkers for PSMA radioligand therapy in mCRPC.

Study name	Year	Study design	Sample size	Treatment context	ctDNA biomarker	Key findings
LuPIN (NOX66 + Lu-PSMA-617) (Crumbaker et al., 2023)	2023	Phase I/II single-arm combination trial	60 ctDNA samples from 32 mCRPC patients	PSMA-RLT + radiosensitizer (idronoxil)	ctDNA mutations	Alterations in AVPC genes (*TP53, RB1, PTEN*) in ctDNA were strongly associated with shorter PSA PFS and OS
Sartor et al. (2023)	2023	Observational, single-institution cohort (retrospective/prospective biomarker) study	44 patients with mCRPC	PSMA-RLT	ctDNA mutations	*FGFR1, CCNE1,* and *CDK12* may be biomarkers of resistance
PSMAfore (De Bono et al., 2025)	2025	Phase III randomized, open-label trial	468 mCRPC patients (234–177LuPSMA arm vs. 234 ARPi-change arm)	RLT vs. ARPi change (taxane-naïve)	ctDNA fraction dynamics	The C2D1 ctDNA fraction and its drop predict rPFS/OS more accurately than baseline ctDNA and PSA
TheraP (Kwan et al., 2025)	2025	Randomized, open-label Phase II trial	Post hoc analysis of 290 plasma cfDNA samples from 180 mCRPC patients	PSMA-RLT vs. cabazitaxel	ctDNA dynamics and mutations	Low baseline and week 6 ctDNA predict longer PFS/OS and greater Lu-PSMA benefit Demonstrated differential genomic predictors for RLT vs. chemotherapy

## 4 Challenges and limitations of ctDNA in mCRPC

Despite the substantial translational promise of ctDNA in mCRPC, important biological, analytical, and clinical limitations currently restrict its routine implementation. Variability in biological shedding remains a fundamental constraint. Tumor fraction in plasma reflects a complex interplay between tumor burden, vascularity, apoptotic rate, and metastatic site. Bone-predominant disease, characteristic of mCRPC, may shed lower levels of ctDNA than visceral metastases, increasing the risk of false-negative findings in patients with clinically significant disease (Fonseca et al., 2024). Although technological advances such as ultra-low-pass whole-genome sequencing, fragmentomics, and methylation-based assays have improved detection sensitivity to approximately 0.1%–0.5% under optimized conditions (Tébar-Martínez et al., 2023; Postel et al., 2018), biological non-shedding remains an unresolved limitation. A non-detectable ctDNA result, therefore, does not exclude active disease.

Assay heterogeneity and the absence of standardized ctDNA thresholds remain major barriers to clinical implementation in mCRPC. Current platforms differ in gene coverage, tumor-fraction estimation, sequencing depth, error suppression, bioinformatic pipelines, and pre-analytical handling, limiting comparability across studies. Although thresholds such as undetectable, 2%–30%, and >30% ctDNA fraction, or RLT-specific cut-offs such as >0.5% and <2%, have shown prognostic value, they remain assay-specific and context-dependent (De Bono et al., 2025; Kwan et al., 2025; Annala et al., 2018). Importantly, ctDNA tumor fraction is a continuous biological variable, and unvalidated dichotomization into “high” or “low” categories may oversimplify interpretation. Until large, prospective, randomized phase III trials validate clinically meaningful ctDNA response and progression thresholds, supported by harmonized reporting standards and locked analytical pipelines, ctDNA cut-offs should be used primarily for risk stratification rather than routine treatment decision-making (Mateo et al., 2018; Franceschini et al., 2024).

Biological and technical confounders also complicate interpretation. CHIP represents a major source of false-positive mutations in genes such as *TP53*, *DNMT3A*, *TET2,* and *ASXL1,* necessitating matched white blood cell sequencing and rigorous bioinformatic filtering (Mayrhofer et al., 2018). Pre-analytical variability, including tube type, processing delays, and storage conditions, may introduce additional inconsistencies. Furthermore, technical artifacts and contamination can produce misleading signals if robust quality-control measures are not applied. Importantly, ctDNA provides a composite genomic signal derived from multiple metastatic sites but cannot resolve intratumoural architecture or microenvironmental context.

Population-level validation remains another important limitation. Several PCa LB assays have been evaluated predominantly in selected geographic or ethnic cohorts, and the work of Matuszczak et al. highlights that some tests lack adequate validation in African, Asian, Latino, or other diverse populations. Even when multiethnic data are available, optimal thresholds may differ across groups (Matuszczak et al., 2021). This is relevant to ctDNA because tumor fraction thresholds, variant interpretation, CHIP prevalence, germline background, and metastatic patterns may vary across populations. Therefore, prospective ctDNA studies should include diverse cohorts and should avoid assuming that thresholds derived from one population or assay platform are universally applicable.

Resistance mechanisms in mCRPC may involve epigenetic reprogramming, PSMA downregulation, or lineage plasticity, including neuroendocrine differentiation, processes incompletely captured by mutation-based sequencing alone. Analyses of RLT cohorts, such as TheraP, did not identify a single recurrent genomic driver of resistance, underscoring the biological complexity of treatment failure (Kwan et al., 2025). Adjunct approaches, including methylation profiling, fragmentomic analysis, extracellular vesicle analysis, and complementary imaging, may improve the detection of phenotypic transitions but remain investigational.

Finally, cost and accessibility represent practical barriers. Comprehensive longitudinal ctDNA profiling, particularly whole-exome or genome sequencing, is not universally available, and reimbursement for serial monitoring remains limited (Buchanan et al., 2025). Demonstration of health-economic benefit, such as earlier discontinuation of ineffective therapies, will be critical for widespread adoption.

Collectively, these limitations highlight that although ctDNA is one of the most informative LB modalities in mCRPC, its integration into routine care requires prospective validation, assay harmonization, standardized reporting, population-specific evaluation, and regulatory alignment. Rather than functioning as a stand-alone test, ctDNA is best conceptualized as part of a multimodal precision oncology framework that combines tumor-derived genomic information with clinical, biochemical, histopathological, and imaging data.

## 5 Future directions

Several emerging technologies may substantially expand the role of ctDNA as a prognostic and monitoring biomarker in mCRPC, thereby enhancing its potential clinical utility. These approaches aim to improve analytical sensitivity, detect biologically relevant disease states, and overcome limitations associated with low tumour shedding and mutation-based assays. Examples of key emerging ctDNA and cfDNA-based technologies relevant to mCRPC are summarized in Table 5.

**TABLE 5:** Emerging ctDNA technologies relevant to mCRPC.

Technology	Principal advantage	Clinical maturity	References
Fragmentomics	Improved detection at low ctDNA levels; tissue-of-origin information	Advanced research/early clinical development	Cristiano et al. (2019), Mouliere et al. (2018)
cfDNA methylation profiling	Detection of lineage plasticity, neuroendocrine transformation, and tissue-of-origin signals	Advanced clinical development	Lleshi et al. (2024), Berchuck et al. (2022)
Machine learning and AI	Improved classification and integration of complex biomarker datasets	Rapidly expanding	Bruhm et al. (2025), Zhu et al. (2026), Nguyen et al. (2025)
Multi-omic liquid biopsy	Combined genomic, epigenomic, transcriptomic, and phenotypic assessment	Emerging	Casanova-Salas et al. (2021)
Multi-cancer early detection (MCED) concepts	Potential for early detection and tissue-of-origin analysis	Active clinical validation	Bruhm et al. (2025)

Fragmentomics analyses cfDNA fragment size distributions, end motifs, nucleosome positioning, and genome-wide fragmentation patterns. These features can improve ctDNA detection in patients with low tumour fractions and may provide information regarding tissue of origin and chromatin organisation (Mouliere et al., 2018; Cristiano et al., 2019). Recent reviews have highlighted fragmentomics as one of the most promising strategies for enhancing LB sensitivity in PCa and other solid tumours (Bruhm et al., 2025).

DNA methylation analysis represents another important advancement because methylation signatures are tissue-specific, biologically stable, and closely linked to cellular lineage. In PCa, methylation-based assays have demonstrated the ability to identify neuroendocrine differentiation, lineage plasticity, and aggressive disease phenotypes that may not be detected by targeted mutation panels (Berchuck et al., 2022; Beltran et al., 2020; Lleshi et al., 2024) Emerging evidence suggests that methylation profiling may improve early detection and facilitate tissue-of-origin identification (Hitchen et al., 2025; de Abreu et al., 2025).

Multi-omic LB approaches are increasingly being explored to address the biological complexity of mCRPC. While ctDNA provides genomic information, resistance mechanisms may also involve transcriptional changes, altered protein expression, extracellular vesicle signalling, and tumour–microenvironment interactions. Integrating ctDNA with circulating RNA, extracellular vesicles, serum biomarkers, and CTCs may therefore provide a more comprehensive representation of tumour biology (Casanova-Salas et al., 2021). CTCs are particularly complementary because they preserve cellular morphology, protein expression, AR-V7 status, and phenotypic features that cannot be captured by cell-free DNA alone (Casanova-Salas et al., 2021).

Artificial intelligence and machine-learning approaches are increasingly being applied to integrate genomic, epigenomic, and clinical datasets derived from LB analyses. These computational methods may improve signal detection, classification, and risk stratification, although prospective validation and standardisation across platforms remain necessary before clinical implementation (Bruhm et al., 2025; Zhu et al., 2026; Nguyen et al., 2025).

Ultimately, future ctDNA research in mCRPC must move beyond retrospective prognostic associations toward prospective biomarker-driven studies that demonstrate clinical utility. Validation of early ctDNA dynamics, assay harmonisation, and integration with imaging and clinical variables will be essential to determine whether ctDNA-guided therapeutic decisions can improve patient outcomes (de Abreu et al., 2025; Li and Sun, 2024).

## 6 Conclusion

ctDNA is a biologically informative LB tool in mCRPC, with tumor fraction consistently associated with survival across major treatment classes. However, most current evidence supports its role as a prognostic and monitoring biomarker rather than a definitive predictive tool, except in established contexts such as HRR testing. Future clinical implementation will require biomarker-driven trials, assay harmonization, and integration with imaging and clinical variables to show that ctDNA-guided decisions can improve patient outcomes.
